# Evaluation of T2-prepared blood oxygenation level dependent functional magnetic resonance imaging with an event-related task: Hemodynamic response function and reproducibility

**DOI:** 10.3389/fnins.2023.1114045

**Published:** 2023-03-02

**Authors:** Xinyuan Miao, Yinghao Li, Xinyi Zhou, Yu Luo, Adrian G. Paez, Dapeng Liu, Peter C. M. van Zijl, Jun Hua

**Affiliations:** ^1^Neurosection, Division of MRI Research, Russell H. Morgan Department of Radiology and Radiological Science, Johns Hopkins University School of Medicine, Baltimore, MD, United States; ^2^F. M. Kirby Research Center for Functional Brain Imaging, Kennedy Krieger Institute, Baltimore, MD, United States; ^3^Department of Biomedical Engineering, Johns Hopkins University, Baltimore, MD, United States

**Keywords:** T2prep, high-field, resolution, brain, imaging–radiology

## Abstract

T2-prepared (T2prep) blood oxygenation level dependent (BOLD) functional MRI (fMRI) is an alternative fMRI approach developed to mitigate the susceptibility artifacts that are typically observed in brain regions near air-filled cavities, bleeding and calcification, and metallic objects in echo-planar-imaging (EPI) based fMRI images. Here, T2prep BOLD fMRI was evaluated in an event-related paradigm for the first time. Functional experiments were performed using gradient-echo (GRE) EPI, spin-echo (SE) EPI, and T2prep BOLD fMRI during an event-related visual task in 10 healthy human subjects. Each fMRI method was performed with a low (3.4 × 3.4 × 4 mm^3^) and a high (1.5 mm isotropic) spatial resolution on 3T and a high resolution (1.5 mm isotropic) on 7T. Robust activation were detected in the visual cortex with all three fMRI methods. In each group of fMRI scans (3T low resolution, 3T high resolution, and 7T high resolution), GRE EPI showed the highest signal change (ΔS/S), largest full-width-at-half-maximum (FWHM) and longest time-to-peak (TTP) extracted from the hemodynamic response functions (HRF), indicating substantial signal contribution from large draining veins which have longer response times than microvessels. In contrast, T2prep BOLD showed the lowest ΔS/S, smallest FWHM, and shortest TTP, suggesting that T2prep BOLD may have a purer T2-weighted BOLD contrast that is more sensitive to microvessels compared to GRE/SE EPI BOLD. This trend was more obvious in fMRI scans performed with a lower spatial resolution on a lower field (3T with a 3.4 × 3.4 × 4 mm^3^ voxel). Scan-rescan reproducibility in the same subjects was comparable among the three fMRI methods. The results from the current study are expected to be useful to establish T2prep BOLD as a useful alternative fMRI approach for event-related fMRI in brain regions with large susceptibility artifacts.

## Introduction

Blood oxygenation level dependent (BOLD) functional MRI (fMRI) has been the method of choice for numerous brain studies. Whereas functional paradigms with a block design are widely used in various fMRI studies, event-related functional paradigms are more commonly employed in cognitive studies. A number of techniques have been developed for human fMRI. The gradient-echo (GRE) echo-planar-imaging (EPI) is the predominant method for BOLD fMRI. Alternatively, spin-echo (SE) based fMRI methods have been proposed with the expectation that it has a better spatial specificity toward microvessels near the neuronal activation sites albeit a lower contrast compared to GRE EPI BOLD. SE EPI has been the regular pulse sequence adopted for SE-based fMRI. More recently, a whole-brain T2-prepared (T2prep) BOLD fMRI approach (Hua et al., 2014, 2017; Miao et al., 2020, 2021) was proposed, in which a double refocusing T2 preparation module was applied for generating the T2-weighted SE BOLD contrast, followed by a single-shot 3D fast GRE (also known as turbo field echo or TFE) readout. The T2prep BOLD fMRI approach was originally developed on 7T (Hua et al., 2014) in order to mitigate susceptibility artifacts in brain regions near air-filled cavities typically observed in EPI based fMRI images especially on 7T. It was later adapted to 3T and has been applied in patients undergoing presurgical functional mapping suffering from large susceptibility artifacts in the brain resulting from bleeding, metallic surgical hardware and calcification (Hua et al., 2017), as well as in fMRI studies of healthy subjects with metallic head implants (Miao et al., 2020). However, to date, T2prep BOLD fMRI has not been applied in an event-related functional task. In this study, our goal is to evaluate the hemodynamic response functions (HRF) measured using T2prep BOLD fMRI in an event-related functional task and to compare it with the widely used GRE EPI and SE EPI BOLD fMRI methods. Functional experiments using the three fMRI methods were performed with a low and a high spatial resolution on 3T and a high resolution on 7T in healthy human subjects. Hemodynamic response functions (HRF) from respective methods were used as the primary measure to compare and characterize their performance, similar to previous studies with a comparable experimental design (Hulvershorn et al., 2005; Siero et al., 2013). Reproducibility was assessed in the same subjects for all three fMRI methods.

## Materials and methods

### Study participants

Ten healthy subjects (48 ± 13yo, five females) were recruited for the study. We declare that all experiments on human subjects were conducted in accordance with the Declaration of Helsinki. This study was approved by the Johns Hopkins Institutional Review Board, and written informed consent was obtained from each participant. All participants had normal vision and had no history of neurologic or psychiatric disorders.

### MRI scans

All participants were scanned on a 3.0 Tesla (3T) and a 7.0 Tesla (7T) Philips MRI scanner (Philips Healthcare, Best, Netherlands). On 3T, a 32-channel phased-array head coil was used for signal reception and a body coil for transmit. On 7T, a 32-channel phased-array head coil was used for signal reception and an eight-channel transmit head coil for transmit. An advanced B0 shim algorithm was applied using the MRCodeTool software (v1.5.9, TeslaDC, Zaltbommel, Netherlands) installed on the scanner to improve B0 field homogeneity over the entire brain. In order to improve B1 field homogeneity, rectangular pads filled with high dielectric constant materials (Teeuwisse et al., 2012) were placed on the side of the subjects’ head. During fMRI scans, breathing pattern was recorded using a respiratory belt and cardiac pulse was recorded using a finger peripheral pulse unit (PPU) for each participant, so that these parameters can be regressed out for fMRI analysis.

Each participant underwent two fMRI sessions on 3T with a low (3.4 mm) and a high (1.5 mm) spatial resolutions, respectively, and one fMRI session on 7T with a high spatial resolution (1.5 mm). In each fMRI session, the following scans were performed in pseudo-randomized order: (1) Magnetization-Prepared Rapid Gradient-Echo (MPRAGE) for anatomical reference; (2) GRE EPI BOLD fMRI; (3) SE EPI BOLD fMRI; and (4) T2prep BOLD fMRI. Details of all imaging parameters on 3T and 7T are summarized in Table 1. All experiments were repeated on the same participants within 5 days at approximately the same time during the day as the first scan. The imaging slices in the re-scan were carefully placed at approximately the same location as the first scan based on anatomical landmarks (corpus callosum and calcarine fissure). Note that all EPI images were acquired with optimized field homogeneity achieved by higher order shims and image distortions corrected to the best means possible (Schär et al., 2004). Therefore, these images represent the best quality EPI images with least possible dropout and distortion on state-of-the-art clinical MRI scanners. On the other hand, only first-order volume shim was applied in T2prep BOLD fMRI (Hua et al., 2014). Vendor-provided standard geometry distortion correction was applied in all scans.

**TABLE 1:** Imaging parameters in all magnetic resonance imaging (MRI) sequences used in this study.

	3T		7T
MPRAGE	TR/TE/TI = 8.1/3.7/1,100 ms, shot interval = 2,100 ms, voxel = 1 × 1 × 1 mm^3^, 160 slices		TR/TE/TI = 3.6/1.8/563 ms, shot interval = 4,500 ms, voxel = 1 × 1 × 1 mm^3^, 180 slices
**BOLD fMRI**			
**Common parameters: FOV = 210 × 210 mm^2^, TR = 2 s**			
Voxel	3.4 × 3.4 × 3 mm^3^	1.5 × 1.5 × 1.5 mm^3^	1.5 × 1.5 × 1.5 mm^3^
GRE-EPI	TR/TE = 2,000/35 ms, FA = 90^°^, 10 slices, SENSE factor = 1.8	TR/TE = 2,000/35 ms, FA = 90^°^, 10 slices, SENSE factor = 1.8	TR/TE = 2,000/25 ms, FA = 60°, 10 slices, SENSE factor = 2.7
SE-EPI	TR/TE = 2,000/70 ms, FA = 90^°^, 10 slices, SENSE factor = 1.8	TR/TE = 2,000/70 ms, FA = 90°, 10 slices, SENSE factor = 1.8	TR/TE = 2,000/50 ms, FA = 60°, 10 slices, SENSE factor = 2.7
T2prep	TR/TE (T2prep effective) = 2,000/70 ms, FA = 20°, 10 slices, SENSE factor = 2 (P) × 1.5(Z), TR_GRE_/TE_GRE_ = 3.6/1.7 ms k-space profile: centric (low-high)	TR/TE (T2prep effective) = 2,000/70 ms, FA = 20°, 10 slices, SENSE factor = 2 (P) × 1.5(Z), TR_GRE_/TE_GRE_ = 3.6/1.7 ms k-space profile: centric (low-high)	TR/TE (T2prep effective) = 2,000/50 ms, FA = 7°, 10 slices, SENSE factor = 2 (P) × 1.5(Z), TR_GRE_/TE_GRE_ = 3.6/1.7 ms k-space profile: centric (low-high)

FOV, field of view; TR, time of repetition; TE, time of echo; TI, time of inversion; FA, flip angle.

### Event-related visual stimulation paradigm

During each fMRI scan, an event-related visual stimulation paradigm of flashing checkerboard (radial, red-and-black, flickering at 8 Hz) with a randomized inter-stimulus-interval (ISI) design (Burock et al., 1998) was performed. Visual stimuli of 1 s duration were presented with the ISI set to be randomized odd numbers between 3 and 17 s, with a mean ISI of 11 s (Dale, 1999; Josephs and Henson, 1999; Miezin et al., 2000; Amaro and Barker, 2006). The durations of each block (stimulus plus ISI) were always multiples of TR (2 s). The total duration of the entire task was 332 s. With a TR of 2 s, 166 fMRI image volumes were collected in each fMRI session. The participants were instructed to press a button when the stimuli are presented. Visual stimuli were presented using a projector at the back of the magnet.

### Data analysis

Image analysis was performed mainly using the statistical parametric mapping (SPM) software package (Version 12, Wellcome Trust Centre for Neuroimaging, London, United Kingdom)^1^ and other in-house code programmed in Matlab (MathWorks, Natick, MA, USA). Routines in the SPM software were used for the following preprocessing steps for fMRI data: motion correction using the realignment routine in SPM, slice timing correction (for 2D multi-slice EPI BOLD scans only, not needed for 3D T2prep BOLD scans), co-registration between fMRI and anatomical (MPRAGE) images, segmentation, and normalization to the Montreal Neurological Institute (MNI) space. The visual regions were identified using the IBASPM116 atlas after segmentation and normalization in SPM. No spatial smoothing was applied. A general linear model (GLM) was employed for activation detection (adjusted *P* < 0.05). Motion parameters estimated from the realignment routine (translational movement in the x, y, or z plane and rotation in the yaw, pitch, or roll direction), cardiac pulse and breathing pattern recorded for each participant were regressed out as covariates in the GLM. Activated voxels identified from the GRE EPI, SE EPI, and T2prep BOLD fMRI scans in each fMRI session were combined for further analysis. Time courses from the combined voxels were extracted for each scan. Finite impulse response (FIR) based HRF modeling (Lindquist et al., 2009) was adopted and the HRF were estimated using the HRF estimation toolbox software.^2^ Key HRF parameters, including relative signal change (ΔS/S), full-width-at-half-maximum (FWHM), time-to-peak (TTP) were estimated from the HRF curves using the respective functions in the HRF estimation toolbox software.

One-way analysis of variance (ANOVA) was performed for group comparisons among the three fMRI methods. Multiple comparisons were corrected using the false discovery rate.

To assess the reproducibility, fMRI images from the first and second scans were co-registered using the SPM software. Only slices that were completely covered in both scans after co-registration were included for further analysis. Dice coefficients between the maps of activated voxels from the scan and re-scan of the same subjects were calculated to evaluate the reproducibility of spatial locations of the activated clusters (Sair et al., 2016). In addition, intraclass correlation coefficient (ICC) was calculated to evaluate the reproducibility of HRF parameters between the scan and re-scan results in the same subjects. The definition of ICC in a textbook (Rosner, 2011) was adopted: σ_*A*_^2^/(σ_*A*_^2^ + σ^2^), where σ_*A*_^2^ and σ^2^ are the between-subject and within-subject variance, respectively. The procedure described in the paragraph is identical to that in our previous reproducibility studies (Landman et al., 2011; Miao et al., 2021).

## Results

**FIGURE 1:**
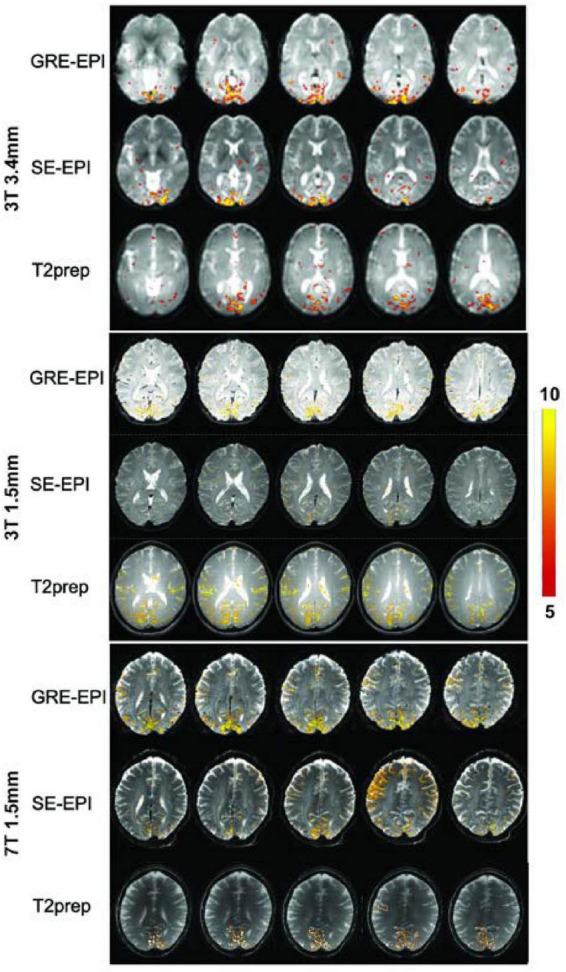
Representative activation maps measured by gradient-echo (GRE) echo-planar-imaging (EPI), spin-echo (SE) EPI, and T2-prepared (T2prep) blood oxygenation level dependent (BOLD) functional MRI (fMRI) on 3T and 7T. The t-values of activated voxels were overlaid on respective fMRI images from each scan. The scale of t-values is indicated by the color bar on the right.

Figure 1 shows representative activation maps measured by GRE EPI, SE EPI, and T2prep BOLD fMRI on 3T and 7T. Similar activations in the visual cortex were detected from all participants. Several subjects also showed activation in some motor regions, as a button was pressed every time the visual stimuli were presented. However, as the purpose of button pressing is to ensure the proper reception of visual stimuli, the pressing action itself can vary substantially. Therefore, the activation detected in the motor regions were not consistent among scans and subjects. Within the visual regions, the activation pattern was more consistent. GRE EPI scans showed larger activation areas than SE EPI and T2prep BOLD, while the activation areas were comparable between SE EPI and T2prep BOLD in most subjects. More than 75% of the activated voxels in visual regions in T2prep BOLD scans overlapped with activated voxels from the other two scans: 80 ± 11% for 3T with a 3.4 × 3.4 × 4 mm^3^ voxel, 75 ± 12% for 3T with a 1.5 mm isotropic voxel, and 76 ± 10% for 7T with a 1.5 mm isotropic voxel. The subsequent analysis focuses on the visual regions.

**FIGURE 2:**
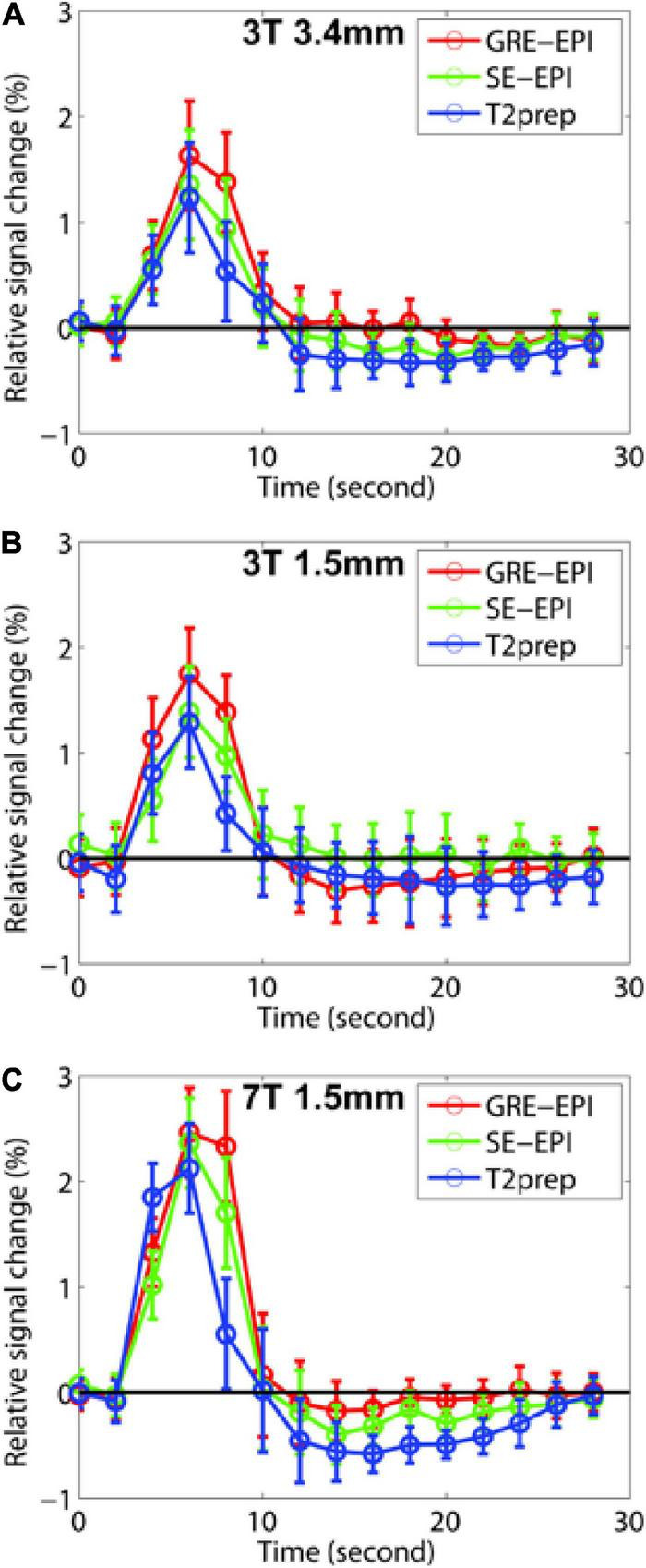
The fitted hemodynamic response functions (HRF) averaged across all participants (*n* = 10) from gradient-echo (GRE) echo-planar-imaging (EPI) (red), spin-echo (SE) EPI (green), and T2-prepared (T2prep) blood oxygenation level dependent (BOLD) functional MRI (fMRI) (blue) on 3T and 7T are shown. Results from 3T with 3.4 × 3.4 × 4 mm^3^ voxel, 3T with 1.5 mm isotropic voxel, and 7T with 1.5 mm isotropic voxel are shown in **(A–C)**, respectively. The error bars indicate inter-subject standard errors.

Figure 2 shows the HRF curves averaged over all subjects (*n* = 10). Table 2 summarizes and compares the key parameters fitted from the HRF curves. In all three fMRI sessions (3T with a 3.4 × 3.4 × 4 mm^3^ voxel, 3T with a 1.5 mm isotropic voxel, and 7T with a 1.5 mm isotropic voxel), GRE EPI showed the highest signal change (ΔS/S), largest FWHM, and longest TTP, whereas T2prep BOLD showed the lowest ΔS/S, smallest FWHM, and shortest TTP. This trend was more significant in fMRI scans performed with a lower spatial resolution (3T with a 3.4 × 3.4 × 4 mm^3^ voxel). In fMRI scans performed with a higher spatial resolution (1.5 mm isotropic voxel) on 3T, ΔS/S was greater, and FWHM and TTP were smaller than respective scans with a 3.4 × 3.4 × 4 mm^3^ voxel (*P* < 0.05). When comparing fMRI scans with a 1.5 mm isotropic voxel performed on 3T and 7T, the 7T scans showed greater ΔS/S, and smaller FWHM and TTP values in respective fMRI methods (**P** < 0.05), and the difference among the three fMRI methods became less significant than 3T.

**TABLE 2:** Quantitative comparison of hemodynamic response functions (HRF) among three blood oxygenation level dependent (BOLD) functional magnetic resonance imaging (fMRI) methods averaged across all subjects on 3T and 7T (*n* = 10).

					*P* ^†⁣†^			
		**GRE EPI**	**SE EPI**	**T2prep**	**All**	**a vs. b**	**a vs. c**	**b vs. c**
3T 3.4 mm	ΔS/S (%)^†^	1.63 ± 0.52	1.40 ± 0.37	1.23 ± 0.21	0.04*	0.05*	0.04*	0.05*
	FWHM (s)	5.34 ± 0.75	4.87 ± 0.90	4.55 ± 0.88	0.03*	0.05*	0.03*	0.05*
	TTP (s)	6.94 ± 0.42	6.10 ± 0.52	5.22 ± 0.53	0.02*	0.03*	0.02*	0.04*
3T 1.5 mm	ΔS/S (%)	1.75 ± 0.38	1.31 ± 0.27	1.29 ± 0.28	0.04*	0.05*	0.04*	0.10
	FWHM (s)	4.97 ± 0.85	4.64 ± 0.72	4.22 ± 0.76	0.05*	0.05*	0.05*	0.05*
	TTP (s)	6.48 ± 0.49	5.81 ± 2.21	5.07 ± 0.59	0.04*	0.05*	0.04*	0.05*
7T 1.5 mm	ΔS/S (%)	2.56 ± 0.33	2.42 ± 0.35	2.21 ± 0.36	0.05*	0.08	0.05*	0.06
	FWHM (s)	4.82 ± 0.69	4.53 ± 0.83	4.16 ± 0.68	0.08	N/A	N/A	N/A
	TTP (s)	6.22 ± 0.74	5.46 ± 0.35	4.94 ± 0.68	0.06	N/A	N/A	N/A
*P* (all)	ΔS/S (%)	0.04*	0.05*	0.05*	–	–	–	–
	FWHM (s)	0.10	0.09	0.09	–	–	–	–
	TTP (s)	0.05*	0.05*	0.08	–	–	–	–
*P* (3T 3.4 vs. 3T 1.5 mm)	ΔS/S (%)	0.22	0.51	0.46	–	–	–	–
	FWHM (s)	N/A^†⁣†⁣†^	N/A	N/A	–	–	–	–
	TTP (s)	0.07	0.09	N/A	–	–	–	–
*P* (3T 3.4 vs. 7T 1.5 mm)	ΔS/S (%)	0.01*	0.01*	0.01*	–	–	–	–
	FWHM (s)	N/A	N/A	N/A	–	–	–	–
	TTP (s)	0.05*	0.05*	N/A	–	–	–	–
*P* (3T 1.5 vs. 7T 1.5 mm)	ΔS/S (%)	0.01*	0.01*	0.01*	–	–	–	–
	FWHM (s)	N/A	N/A	N/A	–	–	–	–
	TTP (s)	0.07	0.09	N/A	–	–	–	–

Mean ± standard error.

^†^Key hemodynamic response functions (HRF) parameters such as relative signal change (ΔS/S), full-width-at-half-maximum (FWHM), time-to-peak (TTP) were calculated from the HRF curves using the toolbox described in Methods.

^†⁣†^One-way analysis of variance (ANOVA).

^†⁣†⁣†^When the overall *P*-value is not significant, the pairwise *P*-values are not calculated.

*P < 0.05.

Table 3 summarizes the reproducibility results for all three fMRI methods. The spatial locations of activated clusters in the visual cortex showed a good reproducibility with Dice coefficients ranging from 0.76 to 0.80. The fitted HRF parameters showed comparable reproducibility (ICC) to BOLD fMRI measures reported in our previous 3T (Landman et al., 2011) and 7T studies (Miao et al., 2021). No significant difference was found in the reproducibility measures among the three fMRI methods.

**TABLE 3:** Reproducibility of activation maps and hemodynamic response functions (HRF) in gradient-echo (GRE) echo-planar-imaging (EPI), spin-echo (SE) EPI, and T2-prepared (T2prep) blood oxygenation level dependent (BOLD) functional magnetic resonance imaging (fMRI) (*n* = 10).

		GRE-EPI	SE-EPI	T2prep
3T 3.4 mm	Dice^†^ coefficient	0.76 ± 0.07	0.77 ± 0.08	0.80 ± 0.06
	ICC^†⁣†^ of ΔS/S	0.80 ± 0.09	0.79 ± 0.08	0.83 ± 0.09
	ICC of FWHM	0.83 ± 0.05	0.80 ± 0.10	0.80 ± 0.06
	ICC of TTP	0.80 ± 0.11	0.80 ± 0.12	0.77 ± 0.07
3T 1.5 mm	Dice coefficient	0.76 ± 0.11	0.77 ± 0.10	0.79 ± 0.09
	ICC of ΔS/S	0.80 ± 0.10	0.79 ± 0.09	0.81 ± 0.09
	ICC of FWHM	0.80 ± 0.09	0.81 ± 0.07	0.83 ± 0.09
	ICC of TTP	0.76 ± 0.08	0.80 ± 0.11	0.85 ± 0.11
7T 1.5 mm	Dice coefficient	0.76 ± 0.07	0.77 ± 0.11	0.78 ± 0.06
	ICC of ΔS/S	0.82 ± 0.06	0.79 ± 0.10	0.80 ± 0.06
	ICC of FWHM	0.81 ± 0.11	0.82 ± 0.09	0.80 ± 0.05
	ICC of TTP	0.76 ± 0.12	0.80 ± 0.08	0.82 ± 0.08

Mean ± standard error.

^†^Dice coefficients between the maps of activated voxels from the scan and re-scan of the same subjects were calculated to evaluate the reproducibility of spatial locations of the activated clusters value. The e of a Dice coefficient ranges from 0, indicating no spatial overlap between the scan and re-scan results, to 1, indicating complete overlap.

^†⁣†^Intraclass correlation coefficient (ICC) between the scan and re-scan results in the same subjects.

## Discussions

The current study assessed a recently developed T2prep BOLD fMRI approach in an event-related visual task. Functional results from T2prep BOLD fMRI were compared with GRE EPI and SE EPI BOLD fMRI with different spatial resolution on 3T and 7T. Robust activation were detected in the visual cortex with all three fMRI methods. Scan-rescan reproducibility in the same subjects was comparable among the three fMRI methods.

Hemodynamic response functions (HRF) measured from the three fMRI methods showed significant difference. The T2*-weighted BOLD contrast in GRE BOLD originates from both microvessels and large draining veins, while the T2-weighted BOLD contrast in SE BOLD is more sensitive to microvessels (van Zijl et al., 1998; Duong et al., 2003). Thus, first, GRE EPI BOLD showed greater relative signal changes (ΔS/S) than SE EPI and T2prep BOLD. In SE EPI BOLD, only the echo acquired at the exact TE is a pure T2 contrast, while the other echoes still have residual T2*-weighting. Thus, ΔS/S in SE EPI BOLD was slightly larger than ΔS/S in T2prep BOLD. Second, the response and recovery times in large draining veins are longer than those in microvessels. Therefore, GRE EPI BOLD showed a significantly longer TTP and wider FWHM values compared to SE EPI and T2prep BOLD, consistent with previous reports (Hulvershorn et al., 2005; Siero et al., 2013). In a study from Hulvershorn et al. (2005), HRFs from GRE and SE BOLD fMRI were measured with varying TE and a spatial resolution of 3.75 × 3.75 × 4 mm^3^ on 3T, similar to the low resolution fMRI scans performed on 3T in the current study. The TTP for GRE BOLD at TE = 30 ms was significantly longer than SE BOLD at TE = 70 ms, congruent with our results. Among the three methods, T2prep BOLD showed the shortest TTP and smallest FWHM, indicating a purer T2-weighted BOLD contrast compared to the other two fMRI methods. Such difference was more significant with a lower spatial resolution on 3T. When acquiring with a higher spatial resolution, ΔS/S became greater and TTP and FWHM became shorter in respective fMRI methods, and the difference among the three fMRI methods became less significant, which may be attributed primarily to the reduced partial volume effects (Siero et al., 2013). At higher magnetic fields (7T), extravascular effects became more dominant in both GRE and SE BOLD (Siero et al., 2013; Cheng et al., 2015). Our results showed that ΔS/S further increased while TTP and FWHM decreased further in respective fMRI methods mainly due to the shorter response and recovery times in microvessels, and the difference among the three fMRI methods became less significant compared to fMRI scans performed with the same spatial resolution (1.5 mm isotropic) on 3T. In a study from Siero et al. (2013), HRFs from GRE and SE BOLD fMRI were measured with a spatial resolution of 1 mm isotropic and 2 mm isotropic, respectively on 7T, similar to the 7T fMRI scans performed in the current study. The relative signal change ΔS/S was smaller in SE BOLD while TTP and FWHM were both shorter in SE BOLD compared to GRE BOLD, all of which align with the results in the current study. One limitation of the current study is that the temporal resolution of fMRI scans was not sufficient to measure the onset time of BOLD signals, which may be more sensitive to the difference between microvessels and large draining veins. Subsequent studies are warranted to evaluate the onset time differences in these scans.

One main difference between the T2prep BOLD and GRE/SE EPI fMRI methods lies in the timing when the BOLD contrast is generated during the sequence. In the 2D multi-slice GRE/SE EPI BOLD sequences, the BOLD contrast is generated during the entire readout period. On the other hand, T2prep BOLD is a 3D sequence, in which the BOLD contrast is induced by the T2-preparation module lasting for about 50 ms at the beginning of each TR, while the readout pulse train during the rest of the volume TR has little T2/T2* weighting with a very short TE (<3 ms). This has essential implication for the design of functional experiments using these approaches. When using T2prep BOLD fMRI, the functional stimulus needs to occur before or to coincide with that start of the T2-preparation module in order to properly induce the BOLD contrast. In our experiments, odd ISI values with visual stimuli of 1 s were used in order to synchronize the onset of stimuli with the beginning of the TR (2 s). This technical consideration is particularly important for event-related paradigms.

## Conclusion

In this study, the recently developed T2prep BOLD fMRI approach was evaluated in an event-related functional paradigm for the first time. Compared to the conventional GRE EPI and SE EPI BOLD fMRI methods, T2prep BOLD demonstrated comparable functional results including activation maps and scan-rescan reproducibility using an event-related visual task on both 3T and 7T. The HRFs extracted from the fMRI data suggest that T2prep BOLD may have a purer T2-weighted BOLD contrast that is more sensitive to microvessels compared to GRE/SE EPI BOLD. The main advantage of T2prep BOLD compared to EPI based fMRI methods is that it can provide clear access to brain regions that suffer from large susceptibility artifacts in conventional EPI scans. Therefore, the results from the current study are expected to be useful for future fMRI studies that need to use T2prep BOLD fMRI in such brain regions with an event-related design.

## Data availability statement

The raw data supporting the conclusions of this article will be made available by the authors, without undue reservation.

## Ethics statement

The studies involving human participants were reviewed and approved by Johns Hopkins Institutional Review Board. The patients/participants provided their written informed consent to participate in this study.

## Author contributions

XM and YLi: organization and execution of the study, statistical analysis, writing of the manuscript, and review and critique of the manuscript. XZ, YLu, and AP: organization and execution of the study and review and critique of the manuscript. DL and PZ: review and critique of the manuscript. JH: conception and design, organization and execution of the study, statistical analysis, writing of the manuscript, and review and critique of the manuscript. All authors contributed to the article and approved the submitted version.

## Footnotes

1
http://www.fil.ion.ucl.ac.uk/spm/

2
https://github.com/canlab/CanlabCore/tree/master/CanlabCore/HRF_Est_Toolbox2
